# Quantitative trait locus analysis for pod- and kernel-related traits in the cultivated peanut (*Arachis hypogaea* L.)

**DOI:** 10.1186/s12863-016-0337-x

**Published:** 2016-01-25

**Authors:** Weigang Chen, Yongqing Jiao, Liangqiang Cheng, Li Huang, Boshou Liao, Mei Tang, Xiaoping Ren, Xiaojing Zhou, Yuning Chen, Huifang Jiang

**Affiliations:** Key Laboratory of Biology and Genetic Improvement of Oil Crops, Ministry of Agriculture, Oil Crops Research Institute of the Chinese Academy of Agricultural Sciences, Wuhan, 430062 China

**Keywords:** Peanut (*Arachis hypogaea L.*), QTL analysis, Pod length, Pod width, Seed length, Seed width

## Abstract

**Background:**

The cultivated peanut (*Arachis hypogaea* L.) is an important oil and food crop in the world. Pod- and kernel-related traits are direct factors involved in determining the yield of the peanut. However, the genetic basis underlying pod- and kernel-related traits in the peanut remained largely unknown, which hampered the improvement of peanut through marker-assisted selection. To understand the genetic basis underlying pod- and kernel-related traits in the peanut and provide more useful information for marker-assisted breeding, we conducted quantitative trait locus (QTL) analysis for pod length and width and seed length and width by use of two F_2:3_ populations derived from cultivar Fuchuan Dahuasheng × ICG 6375 (FI population) and cultivar Xuhua 13 × cultivar Zhonghua 6 (XZ population) in this study.

**Results:**

Two genetic maps containing 347 and 228 polymorphic markers were constructed for FI and XZ populations respectively. In total, 39 QTLs explaining 1.25–26.11 % of the phenotypic variations were detected in two populations. For the FI population, 26 QTLs were detected between the two environments, among which twelve were not mapped before. For the XZ population, thirteen QTLs were detected, among which eight were not reported before. One QTL for pod width was repeatedly mapped between the two populations.

**Conclusion:**

The QTL analyses for pod length and width and seed length and width were conducted in this study using two mapping populations. Novel QTLs were identified, which included two for pod length, four for pod width, five for seed length and one for seed width in the FI population, and three for pod length, three for pod width and two for seed width in the XZ population. Our results will be helpful for improving pod- and seed-related traits in peanuts through marker-assisted selection.

**Electronic supplementary material:**

The online version of this article (doi:10.1186/s12863-016-0337-x) contains supplementary material, which is available to authorized users.

## Background

The cultivated peanut (*Arachis hypogaea* L.), also known as groundnut, is an allotetraploid (2n = 4x =40) legume that is widely grown in semi-arid regions in the world as an important oil or food crop. In 2013, the global yield of the peanut was estimated to be 45.65 million tonnes [1]. The actual yield of peanut cultivars in the farmers’ fields is far below their yield potential. Breeding peanut cultivars with a high yield is one of major objectives in peanut-breeding programs. Pod- and kernel-related traits are direct factors involved in yield determination [2]. The improvement of pod- and kernel-related traits is important for the development of peanut cultivars with a high yield performance.

Quantitative trait locus (QTL) mapping has been widely conducted for various crops to detect the genomic regions controlling important agronomic traits [3–7]. By use of this method, molecular markers tightly linked to the QTL can be developed and further deployed in marker-assisted breeding to improve the efficiency of conventional breeding. Due to the low level of genetic diversity of the peanut germplasm [8], QTL mapping in the peanut has been slow in the past. In recent years, progress has been achieved in the molecular mapping of the peanut. Gomez Selvaraj et al. [9] reported five SSR markers that were associated with seed length, pod length, number of pods per plant, 100-seed weight, maturity and oil content by use of a bulked segregant analysis. Despite non-classical QTL mapping with genetic linkage map, this paper is the first report to attempt to identify QTLs controlling pod- and kernel-related traits in the cultivated peanut. Khedikar et al. [10] mapped 11 QTLs for late leaf spot (LLs) and 12 for rust by use of a recombinant inbred line (RIL) population. Wang et al. [11] reported 23 QTLs, which included one for thrips, nine for tomato spotted wilt virus (TSWV), and thirteen for LLS through a RIL population derived from the cultivar Tifrunner × GT-C20. In a study focusing on drought tolerance related traits, Ravi et al. [12] reported 53 main-effect and 8 epistatic QTLs, among which four main-effect QTLs for pod weight and two main-effect QTLs for seed weight were identified. Shirasawa et al. [13] identified a total of 23 QTLs for different agronomic traits, including twelve QTLs for pod- and seed-related traits. In these two papers, the QTLs for pod weight, pod length and seed weight were commonly mapped on linkage group (LG) A5.

Although some QTLs associated with pod and seed related traits were reported [2, 12], more loci need to be identified to provide helpful information for marker-assisted selection in peanut breeding. Compared to ICG 6375 and cultivar (cv) Zhonghua 6, cv Fuchuan Dahuasheng and cv Xuhua 13 had larger pods and kernels. The objectives of this study were to identify QTLs controlling pod length (PL) and width (PW) and seed length (SL) and width (SW) in cv Fuchuan Dahuasheng and cv Xuhua 13 through two F_2:3_ populations derived from cv Fuchuan Dahuasheng × ICG 6375 and cv Xuhua 13 × cv Zhonghua 6, respectively.

## Results

### Phenotypic variation

**Fig. 1:**
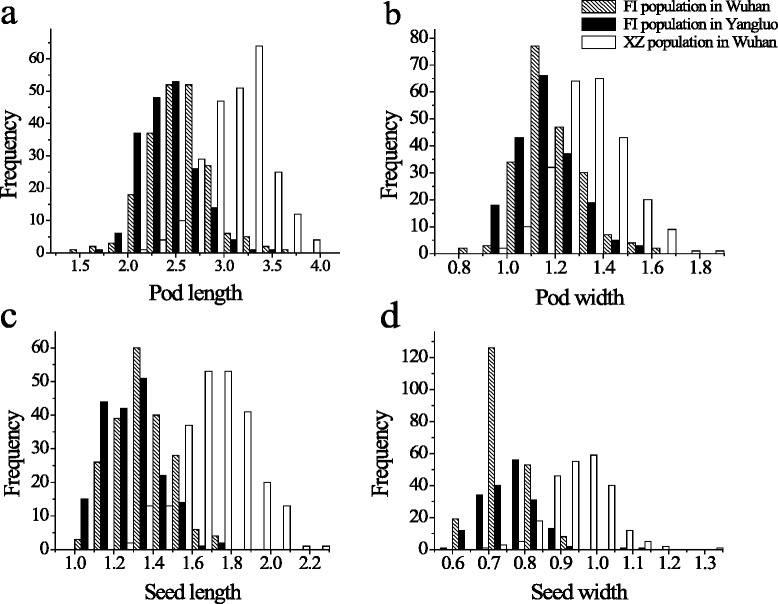
Distribution of pod length, pod width, seed length and seed width. **a** distribution of pod length, **b** distribution of pod width, **c** distribution of seed length, **d** distribution of seed width

The pod length and width and the seed length and width were evaluated in the FI and XZ populations. Both of the populations showed a large genetic variation in these four traits among the F_2:3_ progenies (Tables 1, 2, Fig. 1). The normality test by the Shapiro–Wilk (*w*) indicated that the phenotypic data of seed length for the FI population in Wuhan and the phenotypic data of pod length, pod width and seed length for the XZ population were normally distributed, while others were not. The broad-sense heritability of the phenotypic data for each trait was calculated based upon the analysis of variance of family means. The values ranged from 0.63 to 0.93. The Pearson correlation coefficients of the phenotypic data among four traits for the two populations ranged from 0.32 (*P* < 0.0001) between pod length and seed width for the FI population in Wuhan to 0.79 (*P* < 0.0001) between pod length and seed length for the FI population in Wuhan (Table 3).

**Table 1:** Descriptive statistical analysis of the four traits

Pop	Env	Trait	P1	P2	Max (cm)	Min (cm)	Mean (cm)	SD	*H* ^2^	Shapiro-Wilk(*w*)	Kurt	Skew
FI	Wuhan	PL	3.05	1.82	3.66	1.39	2.52	0.33	0.89	0.98(0.03)	1.12	0.27
		PW	1.43	1.01	1.63	0.80	1.15	0.13	0.84	0.96(<0.0001)	1.18	0.75
		SL	1.52	0.96	1.71	0.98	1.31	0.15	0.63	0.99(0.2)	−0.14	0.26
		SW	0.71	0.69	0.94	0.6	0.73	0.07	0.86	0.96(<0.0001)	1.38	0.66
FI	Yangluo	PL	2.93	1.77	3.45	1.65	2.43	0.28	0.98	0.98(0.005)	0.64	0.55
		PW	1.34	0.95	1.59	0.92	1.16	0.12	0.85	0.98(0.002)	0.46	0.56
		SL	1.41	0.98	1.75	1.00	1.29	0.15	0.93	0.98(0.03)	0.01	0.39
		SW	0.75	0.72	1.13	0.59	0.75	0.07	0.77	0.94(<0.0001)	3.72	0.96
XZ	Wuhan	PL	3.60	3.28	3.99	2.19	3.13	0.32	0.82	0.99(0.57)	−0.04	−0.08
		PW	1.50	1.34	1.81	1.01	1.34	0.14	0.79	0.99(0.56)	0.10	0.36
		SL	1.76	1.72	2.27	1.27	1.72	0.17	0.84	0.99(0.36)	−0.04	0.02
		SW	1.08	0.88	1.31	0.74	0.95	0.08	0.81	0.99(0.02)	1.35	0.50

*Pop* Population, *Env* Environments, *P1* female parent, Fuchuan Dahuasheng in FI population and Xuhua 13 in XZ population, *P2* male parent, ICG 6375 in FI population and Zhonghua 6 in XZ population, *PL* pod length, *PW* pod width, *SL* seed length, *SW* seed width, *SD* standard deviation, *H* ^*2*^ broad-sense heritability on entry-mean basis, *Kurt* kurtosis, Skew, skewness

**Table 2:** Variance analysis of the four traits in FI population between Wuhan and Yangluo environment

	Sum of square	*df*	Mean square	*F* value	*P*-value
Pod Length	0.906	1	0.906	10.41	0.001
Pod Width	0.002	1	0.002	0.12	0.730
Seed Length	0.086	1	0.086	4.10	0.044
Seed Width	0.063	1	0.063	12.51	0.000

**Table 3:** Correlation analyses for the four traits

Population	Environment	Phenotype^a^	PL	PW	SL	SW
FI	Wuhan	PL				
		PW	0.62**			
		SL	0.79**	0.58**		
		SW	0.32**	0.53**	0.41**	
FI	Yangluo	PL				
		PW	0.72**			
		SL	0.66**	0.52**		
		SW	0.33**	0.52**	0.55**	
XZ	Wuhan	PL				
		PW	0.71**			
		SL	0.72**	0.66**		
		SW	0.48**	0.66**	0.72**	

** Indicated *P* < 0.01

^a^ *PL* pod length, *PW* pod width, *SL* seed length, *SW* seed width

### Molecular markers and genetic maps

For the FI population, 420 out of 3227 of the SSR markers (Additional file 1) were polymorphic between cv. Fuchuan Dahuasheng and ICG 6375. In total, 347 markers were successfully mapped on 22 LGs, which spanned 1675.6 cM with an average distance of 5.2 cM. The shortest linkage fragment, LG FB4, covering 36.1 cM, had only six markers, and the longest linkage group, LG FA6, had 26 markers, covering 131.5 cM (Table 4, Additional file 2). For the XZ population, 253 out of 2434 of the SSR markers (Additional file 1) were polymorphic between Xuhua 13 and Zhonghua 6. In addition, 228 polymorphic markers were utilized to construct 22 LGs that totally covered a 1337.7 cM genetic distance. The average distance between the adjacent loci was 6.5 cM, and the length of the linkage groups ranged from 21.6 to 111.4 cM (Table 4, Additional file 2).

### QTLs identified in the FI and XZ population

In total, 39 QTLs, explaining 1.25–26.11 % of the phenotypic variations, were detected in the two populations (Table 5). For the FI population, a total of 18 QTLs were detected in the Wuhan environment. Among them, 4 QTLs were detected for pod length with a 5.7–26.11 % phenotypic variation explained (PVE), 6 QTLs for pod width with a 7.42–16.14 % PVE, 5 QTLs for seed length with a 5.66–20.8 % PVE and 3 QTLs for seed width with a 7.42–12.6 % PVE, respectively. The QTLs on LG A5, *qPLA5.1a, qPLA5.1b* and *qPLA5.1c,* together explained more than 24 % of the phenotypic variation for pod length. Other QTLs were detected in the same region, which included *qPLA5.1a* for pod length and *qPWA5.1a* for pod width, *qPLA5.1a* for pod length and *qSLA5.1a* for seed length, *qPLA5.1b* for pod length and *qSLA5.1b* for seed length, *qPLA7.1* for pod length and *qSLA7.1a* for seed length, *qSLA5.1b* for seed length and *qSWA5.1a* for seed width, *qSLA5.1c* for seed length and *qSWA5.1b* for seed width (Table 5, Additional file 3). Eleven QTLs were detected on LG A5, which included 3 for pod length, 3 for pod width, 3 for seed length and 2 for seed width (Table 5, Additional file 3). All of these 11 QTLs were mapped in a similar region on LG FA5 (Table 5).

**Table 4:** Descriptions of the genetic linkage maps of FI and XZ population

FI genetic map						XZ genetic map					
LGs^a^	Locus No.^b^	cM	cM/locus	LGs by Shirasawa	Common marker No.	LGs	Locus No.	cM	cM/locus	LGs by Shirasawa	Common marker No.
FA1 (LGF1)	22	84.1	3.8	A01	15	XA1 (LGX5)	11	78.7	7.2	A01	4
FA2 (LGF2)	10	74.9	7.5	A02	5	XA2 (LGX6)	13	57.9	4.5	A02	2
FA3 (LGF3)	16	72.2	4.5	A03	9	XA3 (LGX7)	9	80.7	9.0	A03	5
FA4 (LGF4)	9	74.4	8.3	A04	6	XA4 (LGX8)	20	93.7	4.7	A04	9
FA5 (LGF5)	22	61.2	2.8	A05	15	XA5 (LGX9)	9	60.6	6.7	A05	2
FA6 (LGF6)	13	131.5	10.1	A06	10	XA6 (LGX10)	12	43.6	3.6	A06	5
FA7 (LGF7)	25	72.1	2.9	A07	21	XA7 (LGX11)	17	68.8	4.0	A07	9
FA8 (LGF8)	6	71.8	12.0	A08	4	XA8 (LGX12)	10	94.9	9.5	A08	3
FA9 (LGF9)	17	98.1	5.8	A09	13	XA9 (LGX13)	14	56.5	4.0	A09	5
FA10 (LGF10)	20	71.1	3.6	A10	20	XA10 (LGX14)	10	46.2	4.6	A10	6
FB1 (LGF11)	19	77.4	4.1	B01	17		-	-	-		
FB2 (LGF12)	24	98.3	4.1	B02	21	XB2 (LGX15)	7	36.6	5.2	B02	2
FB3 (LGF13)	20	95.8	4.8	B03	15	XB3 (LGX16)	24	104.0	4.3	B03	7
FB4 (LGF14)	7	36.1	5.2	B04	7	XB4 (LGX17)	5	30.0	6.0	B04	2
FB5 (LGF15)	18	74.5	4.1	B05	16	XB5 (LGX18)	20	111.4	5.6	B05	5
FB6 (LGF16)	19	66.2	3.5	B06	17	XB6 (LGX19)	6	70.7	11.8	B06	2
FB7 (LGF17)	9	75.0	8.3	B07	8		-	-	-		
FB8 (LGF18)	17	93.8	5.5	B08	16		-	-	-		
FB9 (LGF19)	26	72.0	2.8	B09	22	XB9 (LGX20)	3	29.5	9.8	B09	2
FB10 (LGF20)	16	75.3	4.7	B10	15	XB10 (LGX21)	17	29.9	1.8	B10	8
FA7a (LGF21)	6	47.8	8.0	A07	4	XB3a (LGX22)	7	93.0	13.3	B03	2
FB7a (LGF22)	6	52.0	8.7	B07	5	LGX1	3	49.7	16.6		0
	-	-	-			LGX2	4	21.6	5.4		0
	-	-	-			LGX3	4	56.9	14.2		0
	-	-	-			LGX4	3	22.8	7.6		0
Total	347	1675.6	5.7			Total	228	1337.7	7.2		

^a^The initial “F” and “X” represented the FI population and XZ population, respectively

^b^The number of loci on each linkage group

**Table 5:** Positions, effects, and phenotypic variation explained by QTLs for 4 agronomic traits detected in the 2 populations of 2 environments

Population	Environment	Traits	QTL	LG	Position	Marker interval	LOD	Additive effect	Dominant effect	*R* ^2^ (%)
FI	Wuhan	PL	*qPLA5.1a*	FA5	24.51	AHGS1341—pPGPseq9A7	8.47	0.19	−0.09	24.24
			*qPLA5.1b*	FA5	35.41	TC2B9—Ah4-26	9.22	0.18	−0.11	24.29
			*qPLA5.1c*	FA5	47.61	PM45—GNB533-2	6.63	0.17	−0.16	26.11
			*qPLA7.1*	FA7	34.51	AHGS1980—pPGPseq3A1	3.35	0.10	0.00	5.70
		PW	*qPWA3.1*	FA3	38.31	AY232—AHGS0132	3.11	0.03	−0.05	8.49
			*qPWA5.1a*	FA5	22.31	AHGS1904-2—AHGS1341	5.76	0.06	−0.03	16.14
			*qPWA5.1b*	FA5	30.21	ARS715—TC2B9	3.26	0.05	−0.02	9.42
			*qPWA5.1c*	FA5	41.71	Ah4-26—POCR413	2.73	0.03	−0.04	7.50
			*qPWA8.1*	FA8	55.31	ARS120—AHGS2319	3.93	−0.04	0.04	9.85
			*qPWA10.1*	FA10	42.61	AHGS1606—AHGS1566	3.62	0.05	−0.01	7.42
		SL	*qSLA5.1a*	FA5	24.51	AHGS1341—pPGPseq9A7	7.38	0.08	−0.04	20.80
			*qSLA5.1b*	FA5	35.41	TC2B9—Ah4-26	6.70	0.07	−0.04	16.97
			*qSLA5.1c*	FA5	42.71	PM45—GNB533-2	5.98	0.07	−0.05	19.32
			*qSLA7.1a*	FA7	29.41	AHGS1475—pPGPseq3A1	4.36	0.05	−0.04	11.15
			*qSLA7.1b*	FA7	41.31	AHGS2022—AHGS2413	2.53	0.04	−0.02	5.66
		SW	*qSWA5.1a*	FA5	35.41	TC2B9—Ah4-26	2.94	0.01	−0.03	7.42
			*qSWA5.1b*	FA5	42.71	PM45—GNB533-2	3.04	0.02	−0.04	9.43
			*qSWA10.1*	FA10	22.81	AHGS1939-1—TC1G4	3.93	0.02	−0.03	12.60
FI	Yangluo	PL	*qPLA5.2*	FA5	9.71	ARS760—TC6E1-1	2.85	0.08	−0.06	7.92
			*qPLA7.2*	FA7	47.81	AHGS0346—AHGS1692	3.43	0.10	−0.03	8.61
		PW	*qPWA5.2*	FA5	37.41	TC2B9 —Ah4-26	3.66	0.05	0.02	5.16
			*qPWA10.2*	FA10	51.01	GM2084—ARS710	2.95	0.04	−0.03	8.36
		SL	*qSLA10.2a*	FA10	13.01	pPGPseq3E10-1—AHGS1314-2	4.23	0.04	−0.10	12.81
			*qSLA10.2b*	FA10	19.31	AHGS1939-1—AHGS1314-1	4.30	0.06	−0.06	15.75
			*qSLA10.2c*	FA10	26.41	AHGS1314-1—pPGPseq4H11	3.71	0.05	−0.04	12.37
		SW	*qSWA5.2*	FA5	55.11	ARS702—pPGPseq11C8	2.65	−0.02	0.04	14.43
XZ	Wuhan	PL	*qPLA5.3*	XA5	0.01	0—GM1577	3.32	0.09	0.07	1.25
			*qPLA9.3a*	XA9	16.41	EM87—ARS768	3.41	0.11	0.02	4.09
			*qPLA9.3b*	XA9	22.41	AGGS1925—AGGS2572	3.78	0.11	0.01	5.10
			*qPLA9.3c*	XA9	39.41	ARS205—TC1D2	3.83	0.11	−0.04	7.79
		PW	*qPWA5.3*	XA5	0.01	0—GM1577	3.90	0.05	0.01	4.48
			*qPWA8.3*	XA8	93.81	AGGS2186—TC9F10	4.21	0.05	−0.02	8.78
			*qPWA9.3a*	XA9	16.41	EM87—ARS768	3.44	0.05	0.00	5.35
			*qPWA9.3b*	XA9	22.51	AGGS1925—AGGS2572	3.75	0.05	−0.01	6.79
		SL	*qSLA5.3*	XA5	0.01	0—GM1577	2.60	0.05	0.01	3.03
			*qSLA6.3*	XA6	13.41	GC47—ARS816	3.19	−0.06	0.00	4.87
		SW	*qSWA6.3*	XA6	13.41	GC47—ARS816	3.71	−0.03	−0.01	3.77
			*qSWA8.3a*	XA8	78.11	ARS120—AGGS2186	3.67	0.03	0.00	6.44
			*qSWA8.3b*	XA8	91.81	AGGS2186—TC9F10	3.97	0.03	−0.01	9.76

*LG* the linkage group the QTL located in, Marker interval the flanking marker nearest the 95 % confidence interval, *R* ^*2*^ percentage of the phenotypic variation explained by the QTLs

For the FI population in Yangluo, two QTLs were detected for pod length with a 7.92–8.61 % PVE, 2 for pod width with a 5.16–8.36 % PVE, 3 for seed length with a 12.37–15.75 % PVE and 1 for seed width with a 14.43 % PVE. For seed length, *qSLA10.2a*, *qSLA10.2b* and *qSLA10.2c* were mapped in similar regions (Table 5, Additional file 3). For the genetic loci between Wuhan and Yangluo, QTLs *qPWA5.1c* and *qPWA5.2* for pod length*, qPLA5.1a*, *qPLA5.1b*, *qPLA5.1c*, *qPLA7.1*, *qPLA5.2*, *qPLA7.2* for pod length, *qPWA10.1*, *qPWA10.2* for pod width, and *qSWA5.1a*, *qSWA5.1b*, *qSWA5.2* for seed width were repeatedly detected in overlapping or adjacent regions on the same linkage groups.

For the XZ population, 4 QTLs with a 1.25–7.79 % PVE for pod length, 4 QTLs with a 4.48 %–8.78 PVE for pod width, 2 QTLs with a 3.03–4.87 % PVE for seed length and 3 QTLs with a 3.77–9.76 % for seed width were detected. Among these loci, *qPLA5.3* for pod length, *qPWA5.3* for pod width and *qSLA5.3* for seed length were mapped in the same region (Table 5, Additional file 4).

**Fig. 2:**
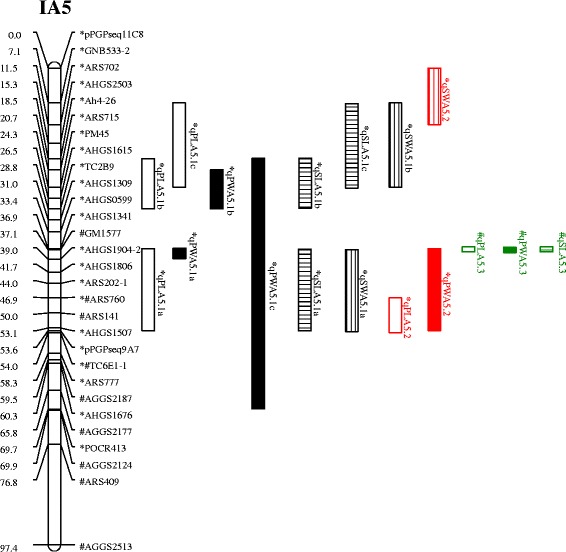
Positions of the QTLs on the integrated genetic linkage of FA5 and XA5. Scale bars on the left side describe the map distance in centimorgans. The markers on the linkage group FA5 of the FI population and XA5 of the XZ population were marked with “*” and “#”, respectively. The common markers between the two populations were marked with both “*” and “#”. The QTLs detected in the FI population were marked with “*”, and the QTLs in Wuhan and Yangluo were shown as black and red, respectively. The QTLs detected in the XZ population were marked with “#” and shown as green. The QTLs for pod length, pod width, seed length, and seed width were filled with none, pure colour, horizontal lines and vertical lines, respectively

To compare the QTLs between the FI and XZ populations, we produced an integrated map because no common markers were linked to the detected QTLs between the two populations (Additional file 2). The results showed that *qPWA5.1a* in the FI population and *qPWA5.3* in the XZ population overlapped on LG IA5 (Fig. 2, Table 5).

## Discussion

The broad-sense heritability estimated in this study was relatively high for pod and seed related traits in the two populations, which means a genetic component, rather than environmental conditions, plays a major role in the determination of these traits. The Pearson correlation coefficient analysis showed that pod length and pod width, as well as pod length and seed length, had a high positive correlation in the two populations, while pod length and seed width had a lower correlation, which was expected. Interestingly, the correlation between seed length and seed width for the FI population was obviously lower than that for the XZ population, which indicates that there may be a different mechanism underlying the determination of seed length and seed width between these two populations.

For both populations, the polymorphic markers were less than ten percent of all of the markers that were evaluated, which made trouble for the production of a high-density linkage map and accurate QTL results. A low level of genetic diversity in the peanut was also reported in other studies [14–16]. Recently, progress had been made on the production of high density linkage maps. Shirasawa et al. [17] constructed a dense integrated map with 3,693 markers on 20 linkage groups spanning 2,651 cM. Zhou et al. [18] construed the first SNP-based genetic linkage map in the cultivated peanut, which was comprised of 1,621 SNPs and 64 SSR markers on 20 linkage groups. Through the use of high-throughput sequencing technology, higher density linkage maps will undoubtedly be expected.

Two or more mapping populations have an advantage over a single population in order to identify more robust QTLs. In our study, two F_2:3_ mapping populations derived from cv Fuchuan Dahuasheng × ICG 6375 (FI) and cultivar Xuhua 13 × cultivar Zhonghua 6 (XZ) were used and the genomic region on the LG A5 were repeatedly mapped between these two populations. Combined with previous studies [12, 13], we believe that LG A5 may harbor important genes for pod and seed related traits, which can be deployed in marker-assisted selection in peanut breeding. Further research, such as fine mapping, should be conducted to investigate the genes responsible for the QTLs on LG A5. For the QTL results in the FI population between the two environments, Wuhan and Yangluo, although QTLs *qPLA5.1a*, *qPLA5.1b*, *qPLA5.1c*, *qPLA7.1*, *qPLA5.2* and *qPLA7.2* for pod length, *qPWA10.1* and *qPWA10.2* for pod width and *qSWA5.1a*, *qSWA5.1b* and *qSWA5.2* for seed width did not overlap, they were mapped to adjacent regions on the same chromosomes. The inconsistency of the QTLs between the two environments might be attributed to the phenotypic deviations caused by environmental factors. QTLs *qPLA7.1*, *qPLA7.2* for pod length, *qPWA3.1*, *qPWA8.1*, *qPWA10.1*, *qPWA10.2* for pod width, *qSLA7.1a*, *qSLA7.1b*, *qSLA10.2a*, *qSLA10.2b*, and *qSLA10.2c* for seed length and *qSWA10.1* for seed width had not been reported before, which provides more valuable sources of loci for the improvement of pod and seed related traits through marker-assisted selection in peanut breeding.

Thirteen QTLs were detected in the XZ population. Eight QTLs, such as *qPLA9.3a*, *qPLA9.3b*, *qPLA9.3c*, *qPWA8.3*, *qPWA9.3a*, *qPWA9.3b*, *qSWA8.3a*, and *qSWA8.3b,* were not reported before and might be novel. Because only phenotypic data from one environment was evaluated, we could not exclude the negative QTL caused by environmental factors. However, compared to the QTL results in the FI population through an integrated map, we found that the QTLs for pod width, *qPWA5.3* in the XZ population and *qPWA5.1a* in FI the population, was repeatedly detected, which indicated that the QTL analyses in the XZ were reliable.

Despite the progress that has been made for genetic mapping in the peanut, QTL analyses are still few in comparison with the research that has been conducted on other crops. The peanut is an important oil or food crop worldwide. More genetic analyses of the genes controlling important agronomic traits, such as yield determining factors and resistance to diseases, will be helpful for improving the peanut through marker-assisted breeding in the future.

## Conclusions

In this study, we conducted QTL analyses for pod and seed related traits in the peanut using two mapping populations, FI and XZ. For the FI population, in total, 26 QTLs were identified in the two environments, among which 12 QTLs were considered as novel loci. For the XZ population, 13 QTLs were detected. One QTL was commonly mapped between the FI and XZ populations. Our results will be helpful for improving pod and seed related traits in peanuts through marker-assisted selection.

## Methods

### Plant materials and phenotypic evaluation

Two F_2_ mapping populations, FI and XZ, were used to construct genetic linkage maps in this study. The FI population was comprised of 218 individuals derived from a cross of Fuchuan Dahuasheng × ICG 6375. Fuchuan Dahuasheng (*A. hypogaea* L. subsp. *hypogaea* L. var. *hirsuta* Kohle) was gathered from Fuchuan County, Guangxi province and is a cultivar with large pods and seeds. ICG 6375 (*A. hypogaea* L. subsp. *fastigata* Waldron var. *vulgaris* Harz) is a groundnut germplasm with small pods received from the International Crop Research Institute for the Semiarid Tropics (ICRISAT). The XZ population was comprised of 282 individuals derived from a cross of cv. Xuhua 13 × cv. Zhonghua 6. Xuhua 13 has large pods and seeds and Zhonghua 6 has small pods and seeds. Xuhua 13 (*A. hypogaea* L. subsp. *hypogaea* L. var*. hypogaea*) is a cultivar developed by the Xuzhou Agricultural Science Research Institute in 2002, and Zhonghua 6 (*A. hypogaea* L. subsp. *fastigata* Waldron var. *vulgaris* Harz.) is a cultivar developed by the Oil Crops Research Institute, Chinese Academy of Agricultural Sciences in 2000. All of the four parents were obtained from the National Mid-term Gene Bank for Oil Crops of China located in Wuhan. In this bank, the accession number of Fuchuan Dahuasheng, ICG 6375, Xuhua 13 and Zhonghua 6 were Zhh 2359, Zhh 7094, Zhh 7778 and Zhh 7629, respectively.

Genomic DNA was extracted from each F_2_ individuals for these two populations following a protocol described by Doyle [19]. The F_2:3_ progenies of each F_2_ individual were harvested and evaluated for pod length, pod width, seed length and seed width. The mean values of each trait of the F_2:3_ progenies were used to represent the phenotype of the F_2_ individuals. For the FI population, the F_2:3_ progenies were grown in two environments, Wuhan in 2012 and Yangluo 2013 in order to evaluate the environmental effects on the traits. Despite only 50 km from each other, the local climate and growing conditions of experimental fields between Wuhan and Yangluo were different. The fields with clay soils in Wuhan were located in the downtown with around 1–2 °C higher than the ones with sandy soil in Yangluo that were located in the countryside. These two experimental fields were owned and managed by Oil Crops Research Institute of the Chinese Academy of Agricultural Sciences. For the XZ population, only Wuhan environment of 2012 was used because of the lack of seeds.

### SSR marker analysis

For each SSR marker, PCR reactions were performed in a T100 Thermal Cycler in a volume of 10 μl, containing 20 ng of DNA template, 0.5 μM of each primer, 10 × PCR buffer, 1 mM MgCl_2_, 0.2 mM dNTPs and 0.5 U Taq polymerase. The PCR temperature profile was 95 °C for 4 min, 35 cycles of 55 s at 94 °C, 45 s at 55 °C and 1 min at 72 °C, and a final extension step of 7 min at 72 °C. The PCR products were visualized on 6 % polyacrylamide gels followed by silver staining. The fragment sizes of the PCR products were estimated by a comparison to a 50 bp DNA ladder.

### Statistical analysis and linkage map

The phenotypic data for pod length and width and seed length and width were tested for normality using the PROC UNIVARIATE procedure of SAS 9.3 (SAS Institute, Cary, NY, USA). The Shapiro–Wilk (*w*) statistic was used to test the null hypothesis that the phenotypic data were normally distributed. Correlation coefficients among the four traits were estimated using the PROC CORR procedure of SAS. The broad-sense heritability for each trait was calculated by a method described by Wu et al. [20].

Genetic linkage maps were constructed using the Joinmap 3.0 software [21] with a maximum recombinant frequency of 0.4. The recombinant ratio was converted to genetic distance by Kossambi map function [22]. The linkage groups were aligned with the reference linkage maps based on the common markers. The Joinmap Combine Groups for Map Integration Module was used to integrate the linkage maps developed in this study.

### QTL mapping

The software Windows QTL Cartographer 2.5 [23] was used to conduct the composite interval mapping (CIM) [24]. The LOD value chosen was 3.4 to declare a QTL significant based on a permutation test [25] with 1,000 runs to determine the *P* = 0.05 genome-wide significance level. To identify more potential QTLs, a QTL with a LOD value more than 2.5 was also presented. The nomenclature of the QTLs was similar to that described by Udall et al. [26] with codes 1 and 2 representing the QTLs detected in the Wuhan and Yangluo environments of the FI population, respectively, and code 3 representing the QTLs detected in the Wuhan environment of the XZ population. If two or more QTLs for the same trait were identified in the same linkage group in the same environment, an alphabetical letter was added at the end of the QTL name. For example, if two QTLs for seed length were detected on A3 in the F_2_ population, they were named as *qSLA3.1a* and *qSLA3.1b*.

### Availability of supporting data

All the supporting data was included as Additional files.

## Additional files

Additional file 1:
**Details of the primer sequences.** (XLSX 33 kb) Additional file 2:
**Information for FI, XZ and the integrated map.** (XLSX 27 kb) Additional file 3:
**Genetic map and positions of the QTLs of the FI population.** Scale bars on the left side described the map distance in centimorgans, and QTLs detected in Wuhan and Yangluo were shown as black and red, respectively. The QTLs for pod length, pod width, seed length, and seed width were indicated by no shading, pure colour, horizontal lines and vertical lines, respectively. The vertical bars on the boxes show the regions over which significant LOD values were calculated by a permutations test (*n* = 1,000). (PPTX 141 kb) Additional file 4:
**Genetic map and positions of the QTLs of the XZ population.** Scale bars on the left side described the map distance in centimorgans, and the QTLs detected in Wuhan and Yangluo were shown as green. The QTLs for pod length, pod width, seed length, and seed width were indicated by no shading, green, horizontal lines and vertical lines, respectively. The vertical bars on the boxes show the regions over which significant LOD values were calculated by a permutations test (*n* = 1,000). (PPTX 108 kb)
