# Supplementary material for: Quantitative trait locus analysis for pod- and kernel-related traits in the cultivated peanut (Arachis hypogaea L.)
Source: BMC Genet. 2016 Jan 25;17:25. doi: 10.1186/s12863-016-0337-x (PMC4727316; doi:10.1186/s12863-016-0337-x)
Supplement: Additional file 3: — Genetic map and positions of the QTLs of the FI population. Scale bars on the left side described the map distance in centimorgans, and QTLs detected in Wuhan and Yangluo were shown as black and red, respectively. The QTLs for pod length, pod width, seed length, and seed width were indicated by no shading, pure colour, horizontal lines and vertical lines, respectively. The vertical bars on the boxes show the regions over which significant LOD values were calculated by a permutations test (n = 1,000). (PPTX 141 kb) [file 12863_2016_337_MOESM3_ESM.pptx]

## Slide 1
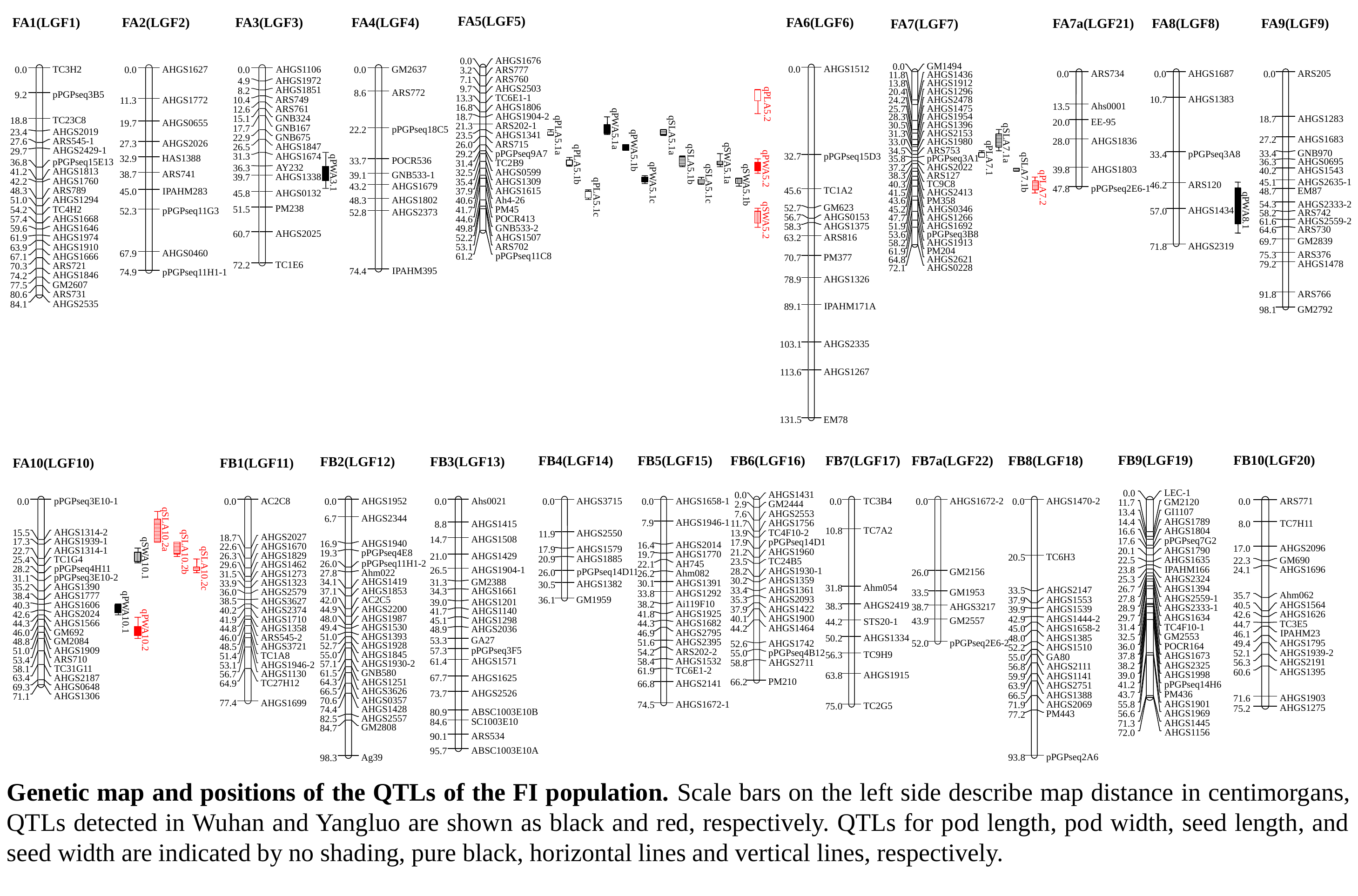

FA5(LGF5)
AHGS1676
0.0
ARS777
3.2
ARS760
7.1
AHGS2503
9.7
TC6E1-1
13.3
qPLA5.2
AHGS1806
16.8
AHGS1904-2
18.7
ARS202-1
21.3
qPWA5.1a
AHGS1341
23.5
qPLA5.1a
qSLA5.1a
ARS715
26.0
qPWA5.1b
pPGPseq9A7
29.2
TC2B9
31.4
qSWA5.1a
qSLA5.1b
qPLA5.1b
qPWA5.2
AHGS0599
32.5
AHGS1309
35.4
qPWA5.1c
qSLA5.1c
qSWA5.1b
AHGS1615
37.9
qPLA5.1c
Ah4-26
40.6
PM45
41.7
POCR413
44.6
qSWA5.2
GNB533-2
49.8
AHGS1507
52.2
ARS702
53.1
pPGPseq11C8
61.2
FA6(LGF6)
AHGS1512
0.0
pPGPseq15D3
32.7
TC1A2
45.6
GM623
52.7
AHGS0153
56.7
AHGS1375
58.3
ARS816
63.2
PM377
70.7
AHGS1326
78.9
IPAHM171A
89.1
AHGS2335
103.1
AHGS1267
113.6
EM78
131.5
FA1(LGF1)
TC3H2
0.0
pPGPseq3B5
9.2
TC23C8
18.8
AHGS2019
23.4
ARS545-1
27.6
AHGS2429-1
29.7
pPGPseq15E13
36.8
AHGS1813
41.2
AHGS1760
42.2
ARS789
48.3
AHGS1294
51.0
TC4H2
54.2
AHGS1668
57.4
AHGS1646
59.6
AHGS1974
61.9
AHGS1910
63.9
AHGS1666
67.1
ARS721
70.3
AHGS1846
74.2
GM2607
77.5
ARS731
80.6
AHGS2535
84.1
FA2(LGF2)
AHGS1627
0.0
AHGS1772
11.3
AHGS0655
19.7
AHGS2026
27.3
HAS1388
32.9
ARS741
38.7
IPAHM283
45.0
pPGPseq11G3
52.3
AHGS0460
67.9
pPGPseq11H1-1
74.9
FA3(LGF3)
AHGS1106
0.0
AHGS1972
4.9
AHGS1851
8.2
ARS749
10.4
ARS761
12.6
GNB324
15.1
GNB167
17.7
GNB675
22.9
AHGS1847
26.5
AHGS1674
31.3
AY232
36.3
qPWA3.1
AHGS1338
39.7
AHGS0132
45.8
PM238
51.5
AHGS2025
60.7
TC1E6
72.2
FA4(LGF4)
GM2637
0.0
ARS772
8.6
pPGPseq18C5
22.2
POCR536
33.7
GNB533-1
39.1
AHGS1679
43.2
AHGS1802
48.3
AHGS2373
52.8
IPAHM395
74.4
FA7a(LGF21)
ARS734
0.0
Ahs0001
13.5
EE-95
20.0
AHGS1836
28.0
AHGS1803
39.8
pPGPseq2E6-1
47.8
FA8(LGF8)
AHGS1687
0.0
AHGS1383
10.7
pPGPseq3A8
33.4
ARS120
46.2
AHGS1434
57.0
qPWA8.1
AHGS2319
71.8
FA9(LGF9)
ARS205
0.0
AHGS1283
18.7
AHGS1683
27.2
GNB970
33.4
AHGS0695
36.3
AHGS1543
40.2
AHGS2635-1
45.1
EM87
48.7
AHGS2333-2
54.3
ARS742
58.2
AHGS2559-2
61.6
ARS730
64.6
GM2839
69.7
ARS376
75.3
AHGS1478
79.2
ARS766
91.8
GM2792
98.1
FA7(LGF7)
GM1494
0.0
AHGS1436
11.8
AHGS1912
13.8
AHGS1296
20.4
AHGS2478
24.2
AHGS1475
25.7
AHGS1954
28.3
AHGS1396
30.5
AHGS2153
31.3
AHGS1980
33.0
qSLA7.1a
ARS753
34.5
pPGPseq3A1
35.8
qPLA7.1
AHGS2022
37.2
qSLA7.1b
ARS127
38.3
TC9C8
40.3
qPLA7.2
AHGS2413
41.5
PM358
43.6
AHGS0346
45.2
AHGS1266
47.7
AHGS1692
51.9
pPGPseq3B8
53.6
AHGS1913
58.2
PM204
61.9
AHGS2621
64.8
AHGS0228
72.1
FB9(LGF19)
LEC-1
0.0
GM2120
11.7
GI1107
13.4
AHGS1789
14.4
AHGS1804
16.6
pPGPseq7G2
17.6
AHGS1790
20.1
AHGS1635
22.5
IPAHM166
23.8
AHGS2324
25.3
AHGS1394
26.7
AHGS2559-1
27.8
AHGS2333-1
28.9
AHGS1634
29.7
TC4F10-1
31.4
GM2553
32.5
POCR164
36.0
AHGS1673
37.8
AHGS2325
38.2
AHGS1998
39.0
pPGPseq14H6
41.2
PM436
43.7
AHGS1901
55.8
AHGS1969
56.6
AHGS1445
71.3
AHGS1156
72.0
FB10(LGF20)
ARS771
0.0
TC7H11
8.0
AHGS2096
17.0
GM690
22.3
AHGS1696
24.1
Ahm062
35.7
AHGS1564
40.5
AHGS1626
42.6
TC3E5
44.7
IPAHM23
46.1
AHGS1795
49.4
AHGS1939-2
52.1
AHGS2191
56.3
AHGS1395
60.6
AHGS1903
71.6
AHGS1275
75.2
FB4(LGF14)
AHGS3715
0.0
AHGS2550
11.9
AHGS1579
17.9
AHGS1885
20.9
pPGPseq14D11
26.0
AHGS1382
30.5
GM1959
36.1
FB5(LGF15)
AHGS1658-1
0.0
AHGS1946-1
7.9
AHGS2014
16.4
AHGS1770
19.7
AH745
22.1
Ahm082
26.2
AHGS1391
30.1
AHGS1292
33.8
Ai119F10
38.2
AHGS1925
41.8
AHGS1682
44.3
AHGS2795
46.9
AHGS2395
51.6
ARS202-2
54.2
AHGS1532
58.4
TC6E1-2
61.9
AHGS2141
66.8
AHGS1672-1
74.5
FB6(LGF16)
AHGS1431
0.0
GM2444
2.9
AHGS2553
7.6
AHGS1756
11.7
TC4F10-2
13.9
pPGPseq14D1
17.9
AHGS1960
21.2
TC24B5
23.5
AHGS1930-1
28.2
AHGS1359
30.2
AHGS1361
33.4
AHGS2093
35.3
AHGS1422
37.9
AHGS1900
40.1
AHGS1464
44.2
AHGS1742
52.6
pPGPseq4B12
55.0
AHGS2711
58.8
PM210
66.2
FB7(LGF17)
TC3B4
0.0
TC7A2
10.8
Ahm054
31.8
AHGS2419
38.3
STS20-1
44.2
AHGS1334
50.2
TC9H9
56.3
AHGS1915
63.8
TC2G5
75.0
FB7a(LGF22)
AHGS1672-2
0.0
GM2156
26.0
GM1953
33.5
AHGS3217
38.7
GM2557
43.9
pPGPseq2E6-2
52.0
FB8(LGF18)
AHGS1470-2
0.0
TC6H3
20.5
AHGS2147
33.5
AHGS1553
37.9
AHGS1539
39.9
AHGS1444-2
42.9
AHGS1658-2
45.0
AHGS1385
48.0
AHGS1510
52.2
GA80
55.0
AHGS2111
56.8
AHGS1141
59.9
AHGS2751
63.9
AHGS1388
66.5
AHGS2069
71.9
PM443
77.2
pPGPseq2A6
93.8
FB2(LGF12)
AHGS1952
0.0
AHGS2344
6.7
AHGS1940
16.9
pPGPseq4E8
19.3
pPGPseq11H1-2
26.0
Ahm022
27.8
AHGS1419
34.1
AHGS1853
37.1
AC2C5
42.0
AHGS2200
44.9
AHGS1987
48.0
AHGS1530
49.4
AHGS1393
51.0
AHGS1928
52.7
AHGS1845
55.0
AHGS1930-2
57.1
GNB580
61.5
AHGS1251
64.3
AHGS3626
66.5
AHGS0357
70.6
AHGS1428
74.4
AHGS2557
82.5
GM2808
84.7
Ag39
98.3
FB3(LGF13)
Ahs0021
0.0
AHGS1415
8.8
AHGS1508
14.7
AHGS1429
21.0
AHGS1904-1
26.5
GM2388
31.3
AHGS1661
34.3
AHGS1201
39.0
AHGS1140
41.7
AHGS1298
45.1
AHGS2036
48.9
GA27
53.3
pPGPseq3F5
57.3
AHGS1571
61.4
AHGS1625
67.7
AHGS2526
73.7
ABSC1003E10B
80.9
SC1003E10
84.6
ARS534
90.1
ABSC1003E10A
95.7
FA10(LGF10)
pPGPseq3E10-1
0.0
qSLA10.2a
AHGS1314-2
15.5
AHGS1939-1
17.3
AHGS1314-1
22.7
qSLA10.2b
qSWA10.1
TC1G4
25.4
pPGPseq4H11
28.2
qSLA10.2c
pPGPseq3E10-2
31.1
AHGS1390
35.2
AHGS1777
38.4
AHGS1606
40.3
qPWA10.1
AHGS2024
42.6
AHGS1566
44.3
qPWA10.2
GM692
46.0
GM2084
48.8
AHGS1909
51.0
ARS710
53.4
TC31G11
58.1
AHGS2187
63.4
AHGS0648
69.3
AHGS1306
71.1
FB1(LGF11)
AC2C8
0.0
AHGS2027
18.7
AHGS1670
22.6
AHGS1829
26.3
AHGS1462
29.6
AHGS1273
31.5
AHGS1323
33.9
AHGS2579
36.0
AHGS3627
38.5
AHGS2374
40.2
AHGS1710
41.9
AHGS1358
44.8
ARS545-2
46.0
AHGS3721
48.5
TC1A8
51.4
AHGS1946-2
53.1
AHGS1130
56.7
TC27H12
64.9
AHGS1699
77.4
Genetic map and positions of the QTLs of the FI population. Scale bars on the left side describe map distance in centimorgans, QTLs detected in Wuhan and Yangluo are shown as black and red, respectively. QTLs for pod length, pod width, seed length, and seed width are indicated by no shading, pure black, horizontal lines and vertical lines, respectively.
