# Supplementary material for: Quantitative trait locus analysis for pod- and kernel-related traits in the cultivated peanut (Arachis hypogaea L.)
Source: BMC Genet. 2016 Jan 25;17:25. doi: 10.1186/s12863-016-0337-x (PMC4727316; doi:10.1186/s12863-016-0337-x)
Supplement: Additional file 4: — Genetic map and positions of the QTLs of the XZ population. Scale bars on the left side described the map distance in centimorgans, and the QTLs detected in Wuhan and Yangluo were shown as green. The QTLs for pod length, pod width, seed length, and seed width were indicated by no shading, green, horizontal lines and vertical lines, respectively. The vertical bars on the boxes show the regions over which significant LOD values were calculated by a permutations test (n = 1,000). (PPTX 108 kb) [file 12863_2016_337_MOESM4_ESM.pptx]

## Slide 1
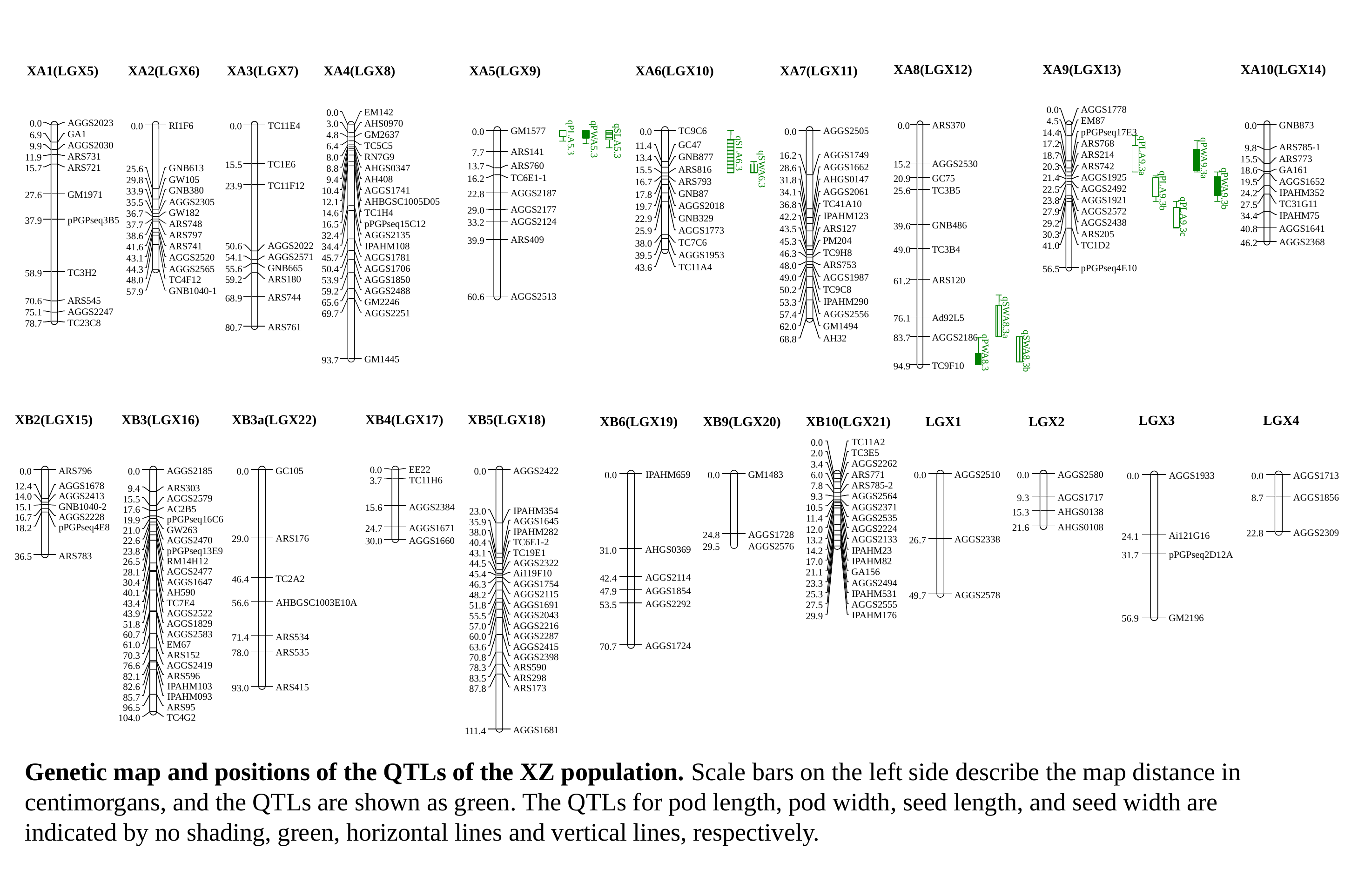

XA8(LGX12)
ARS370
0.0
AGGS2530
15.2
GC75
20.9
TC3B5
25.6
GNB486
39.6
TC3B4
49.0
ARS120
61.2
qSWA8.3a
Ad92L5
76.1
AGGS2186
83.7
qSWA8.3b
qPWA8.3
TC9F10
94.9
XA9(LGX13)
AGGS1778
0.0
EM87
4.5
pPGPseq17E3
14.4
ARS768
17.2
ARS214
qPLA9.3a
18.7
qPWA9.3a
ARS742
20.3
AGGS1925
21.4
qPWA9.3b
AGGS2492
22.5
qPLA9.3b
AGGS1921
23.8
AGGS2572
27.9
qPLA9.3c
AGGS2438
29.2
ARS205
30.3
TC1D2
41.0
pPGPseq4E10
56.5
XA10(LGX14)
GNB873
0.0
ARS785-1
9.8
ARS773
15.5
GA161
18.6
AGGS1652
19.5
IPAHM352
24.2
TC31G11
27.5
IPAHM75
34.4
AGGS1641
40.8
AGGS2368
46.2
XA5(LGX9)
GM1577
0.0
qPLA5.3
qPWA5.3
qSLA5.3
ARS141
7.7
ARS760
13.7
TC6E1-1
16.2
AGGS2187
22.8
AGGS2177
29.0
AGGS2124
33.2
ARS409
39.9
AGGS2513
60.6
XA6(LGX10)
TC9C6
0.0
GC47
11.4
qSLA6.3
GNB877
13.4
qSWA6.3
ARS816
15.5
ARS793
16.7
GNB87
17.8
AGGS2018
19.7
GNB329
22.9
AGGS1773
25.9
TC7C6
38.0
AGGS1953
39.5
TC11A4
43.6
XA7(LGX11)
AGGS2505
0.0
AGGS1749
16.2
AGGS1662
28.6
AHGS0147
31.8
AGGS2061
34.1
TC41A10
36.8
IPAHM123
42.2
ARS127
43.5
PM204
45.3
TC9H8
46.3
ARS753
48.0
AGGS1987
49.0
TC9C8
50.2
IPAHM290
53.3
AGGS2556
57.4
GM1494
62.0
AH32
68.8
XA1(LGX5)
AGGS2023
0.0
GA1
6.9
AGGS2030
9.9
ARS731
11.9
ARS721
15.7
GM1971
27.6
pPGPseq3B5
37.9
TC3H2
58.9
ARS545
70.6
AGGS2247
75.1
TC23C8
78.7
XA2(LGX6)
RI1F6
0.0
GNB613
25.6
GW105
29.8
GNB380
33.9
AGGS2305
35.5
GW182
36.7
ARS748
37.7
ARS797
38.6
ARS741
41.6
AGGS2520
43.1
AGGS2565
44.3
TC4F12
48.0
GNB1040-1
57.9
XA3(LGX7)
TC11E4
0.0
TC1E6
15.5
TC11F12
23.9
AGGS2022
50.6
AGGS2571
54.1
GNB665
55.6
ARS180
59.2
ARS744
68.9
ARS761
80.7
XA4(LGX8)
EM142
0.0
AHS0970
3.0
GM2637
4.8
TC5C5
6.4
RN7G9
8.0
AHGS0347
8.8
AH408
9.4
AGGS1741
10.4
AHBGSC1005D05
12.1
TC1H4
14.6
pPGPseq15C12
16.5
AGGS2135
32.4
IPAHM108
34.4
AGGS1781
45.7
AGGS1706
50.4
AGGS1850
53.9
AGGS2488
59.2
GM2246
65.6
AGGS2251
69.7
GM1445
93.7
XB2(LGX15)
ARS796
0.0
AGGS1678
12.4
AGGS2413
14.0
GNB1040-2
15.1
AGGS2228
16.7
pPGPseq4E8
18.2
ARS783
36.5
XB3(LGX16)
AGGS2185
0.0
ARS303
9.4
AGGS2579
15.5
AC2B5
17.6
pPGPseq16C6
19.9
GW263
21.0
AGGS2470
22.6
pPGPseq13E9
23.8
RM14H12
26.5
AGGS2477
28.1
AGGS1647
30.4
AH590
40.1
TC7E4
43.4
AGGS2522
43.9
AGGS1829
51.8
AGGS2583
60.7
EM67
61.0
ARS152
70.3
AGGS2419
76.6
ARS596
82.1
IPAHM103
82.6
IPAHM093
85.7
ARS95
96.5
TC4G2
104.0
XB3a(LGX22)
GC105
0.0
ARS176
29.0
TC2A2
46.4
AHBGSC1003E10A
56.6
ARS534
71.4
ARS535
78.0
ARS415
93.0
XB4(LGX17)
EE22
0.0
TC11H6
3.7
AGGS2384
15.6
AGGS1671
24.7
AGGS1660
30.0
XB5(LGX18)
AGGS2422
0.0
IPAHM354
23.0
AGGS1645
35.9
IPAHM282
38.0
TC6E1-2
40.4
TC19E1
43.1
AGGS2322
44.5
Ai119F10
45.4
AGGS1754
46.3
AGGS2115
48.2
AGGS1691
51.8
AGGS2043
55.5
AGGS2216
57.0
AGGS2287
60.0
AGGS2415
63.6
AGGS2398
70.8
ARS590
78.3
ARS298
83.5
ARS173
87.8
AGGS1681
111.4
LGX3
AGGS1933
0.0
Ai121G16
24.1
pPGPseq2D12A
31.7
GM2196
56.9
LGX4
AGGS1713
0.0
AGGS1856
8.7
AGGS2309
22.8
XB6(LGX19)
IPAHM659
0.0
AHGS0369
31.0
AGGS2114
42.4
AGGS1854
47.9
AGGS2292
53.5
AGGS1724
70.7
XB9(LGX20)
GM1483
0.0
AGGS1728
24.8
AGGS2576
29.5
XB10(LGX21)
TC11A2
0.0
TC3E5
2.0
AGGS2262
3.4
ARS771
6.0
ARS785-2
7.8
AGGS2564
9.3
AGGS2371
10.5
AGGS2535
11.4
AGGS2224
12.0
AGGS2133
13.2
IPAHM23
14.2
IPAHM82
17.0
GA156
21.1
AGGS2494
23.3
IPAHM531
25.3
AGGS2555
27.5
IPAHM176
29.9
LGX1
AGGS2510
0.0
AGGS2338
26.7
AGGS2578
49.7
LGX2
AGGS2580
0.0
AGGS1717
9.3
AHGS0138
15.3
AHGS0108
21.6
Genetic map and positions of the QTLs of the XZ population. Scale bars on the left side describe the map distance in centimorgans, and the QTLs are shown as green. The QTLs for pod length, pod width, seed length, and seed width are indicated by no shading, green, horizontal lines and vertical lines, respectively.
